# The Problems We Can-SOLVE: How Can-SOLVE CKD Network Implementation and Knowledge Mobilization Projects Are Reshaping Kidney Care in Canada

**DOI:** 10.1177/20543581251333206

**Published:** 2025-04-09

**Authors:** Cathy Woods, Maoliosa Donald, Selina Allu, Michelle Hampson, Cynthia MacDonald, Heather Harris, Malcolm King, James Scholey, Adeera Levin, Matthew T. James

**Affiliations:** 1 Can-SOLVE CKD Network and Department of Medicine, Faculty of Medicine, The University of British Columbia, Winnipeg, Canada; 2 Department of Medicine, O’Brien Institute of Public Health, Cumming School of Medicine, University of Calgary, AB, Canada; 3 Department of Community Health and Epidemiology, University of Saskatchewan, Saskatoon, Canada; 4 Department of Medicine, Faculty of Medicine, University of Toronto, ON, Canada

**Keywords:** knowledge mobilization, implementation science, kidney care

While knowledge on how to treat kidney disease has advanced rapidly in recent years, there is a need to ensure the integration of new evidence into clinical practice. Following a decade-long research endeavor to transform the kidney care landscape as part of the Canadian Institutes of Health Research (CIHR) Strategy for Patient-Oriented Research (SPOR), 9 major kidney health research projects are being integrated into kidney care across Canada. Importantly, patient partners are co-leading this effort, ensuring these transformations in care reflect their values and needs. We are pleased to introduce a 9-part manuscript series describing this journey to deliver better care and outcomes for thousands of Canadians living with kidney disease.

In 2015, the CIHR’s (SPOR) launched a funding opportunity for patient-oriented chronic disease networks.^1^ Canadians Seeking Solutions and Innovations to Overcome Chronic Kidney Disease (Can-SOLVE CKD) was one of 5 chronic disease networks funded. Informed by priority-setting exercises that brought together more than 30 participants, including patients, caregivers, Indigenous peoples, researchers, and policymakers, 18 multidisciplinary research projects were developed, encompassing biomedical first-in-human studies, clinical trials, population health research, and knowledge translation.^2^ This research agenda provided a foundation for the network’s work, beginning a journey to reshape the kidney research landscape across Canada by embedding people with lived experiences with kidney disease into all aspects of kidney research.^3-7^ In the first phase of funding (2016-2021), Can-SOLVE CKD focused on knowledge creation through projects aimed at diagnosing kidney disease earlier, discovering better treatments, and delivering innovative, patient-centered care. Can-SOLVE CKD and its research projects produced 130 peer-reviewed articles, cited more than 2000 times, with 46% of these publications featuring patient partners as co-authors, highlighting a transformative culture shift in kidney research fostered by the network.

One of the most important things that has happened is that we’ve given patients a voice.

*—Cathy Woods, patient partner, Can-SOLVE CKD Network co-lead, from Naicatchewenin Nation*

In response to an opportunity for a second phase of CIHR SPOR network funding, Can-SOLVE CKD shifted to concentrate on moving research knowledge generated from phase 1 into kidney health care policy and practice. Phase 2 Can-SOLVE CKD project proposals were thus developed focusing on implementation science and knowledge mobilization, and were evaluated by patient partners, clinicians, and researchers from across the country, according to 4 major criteria: (1) patient-oriented research program, (2) transformative impact, (3) strength of the team, and (4) feasibility. Based on voting by 36 network members, 9 projects were subsequently selected for the phase 2 grant. In 2021, Can-SOLVE CKD was awarded $3.75 million from CIHR, matched and complimented by more than $8 million of support from >60 partners, to enable these 9 projects to implement, scale-up, and spread innovative kidney care solutions for the millions of Canadians affected by CKD.

Since 2022, Can-SOLVE CKD’s 9 implementation science/knowledge mobilization projects have been advancing implementation of a wide range of initiatives, including treatment and education interventions for vulnerable populations,^8^ evaluation of e-health tools/decision aids,^9^ and clinical trials of new models of providing kidney care.^10,11^ Four network pillars have been promoted across all projects: (1) Implementing, spreading, and scaling up interventions from Can-SOLVE CKD phase one; (2) Advancing Indigenous cultural safety; (3) Diversifying the kidney research environment to address IDEA (Inclusion, Diversity, Equity, Access) principles; and (4) Supporting capacity building and partnerships for sustainable patient-oriented research.

For [patient partners] to be involved in knowledge mobilization in their own community and their own region is vitally important. I think the patients need to be there guiding implementation, because they will do it in a way that’s going to be received well by patients, not just well by doctors and researchers.

—Cathy Woods

Effective and timely implementation of new evidence into health care is a significant challenge. Numerous models and frameworks have been developed to facilitate implementation; however, researchers may be uncertain about which model or framework is most appropriate for their work and uncertain about how to apply implementation models and frameworks. As Can-SOLVE CKD project teams pivoted to focus on implementation science and knowledge mobilization, a strategy to enhance knowledge and capacity in these areas across the network was required. Therefore, multiple levels of support were developed within Can-SOLVE CKD, guided by the Evidence-based System for Innovation Supports (EBSIS) framework.^12^ This framework highlights different types of supports including tools, training, coaching/technical assistance, and an assessment of implementation quality. While this framework was designed to build capacity to increase the adoption and implementation of evidence-based interventions,^13^ it was operationalized within Can-SOLVE CKD it to build capacity and produce tangible resources to support project team to implement their interventions. Specifically, the following tailored supports were put in place:

1. Tools, including the Can-SOLVE CKD Pathway to Implementation guide and tool kit were developed to guide project teams and document implementation sciences/knowledge mobilization approaches and how they would be applied to their projects.^14^
2. Training, including 2 virtual workshops, was provided by The Center for Implementation (TCI) (https://thecenterforimplementation.com/) to 39 network members. In addition, an asynchronous training program in implementation science was offered for all project teams, through 3 online courses from TCI), on “The HOW of Creating Sustainable Change,” “Implementation Scale and Spread,” and “Implementing Community Change.”
3. Technical assistance, consisting of consultative support by experienced Can-SOLVE Knowledge Translation Committee members, including implementation scientists, knowledge brokers, and Senior Renal Program Leaders, was provided to respond to project team questions when required.^15^
4. Continuous quality assurance was maintained by an annual reporting process to the Can-SOLVE CKD Research Operations and Knowledge Translation (ROCKeT) Committee. The annual reports by each project team provided updates on research activities and progress. The ROCKeT Committee then provided specific feedback on knowledge mobilization and implementation plans to the teams on a continuous basis.

There have been many lessons learned during our journey, including important insights into successes and barriers to implementation in kidney care. Thus, we are pleased to offer a special collection of manuscripts in the Canadian Journal of Kidney Health and Disease describing implementation frameworks and resources used, what has proved useful, challenges faced, and strategies used by project team to overcome these challenges to implementing Can-SOLVE CKD innovations for kidney care across Canada. Can-SOLVE CKD phase 2 projects are extending the impact of the network to patients across the country while leading the way to a more inclusive research system that achieves these goals by elevating the patient voice, celebrating diversity, and strengthening cultural competency. We hope this information helps other research teams and clinical programs seeking to implement similar innovations in clinical care in Canada, and globally.

We’ve proved that patient-oriented research works, and I think that that’s going to be our legacy.

*—*Cathy Woods

## Reflections From Patient Partner Cathy Woods

Cathy Woods, of Naicatchewenin First Nation, is an Anishinaabe woman whose traditional name is Giwetashked Giniw Ikewe (Circling Golden Eagle Woman). Woods was first diagnosed with membranous nephropathy at age 57 and considers herself one of the lucky patients whose kidney disease was caught early and cured. Since then, she has dedicated much of her life to improving the lives of people with kidney disease across Turtle Island, through her work as a patient partner with Can-SOLVE CKD.

In 2015 I was asked to attend a prioritization meeting for kidney projects that were on the horizon. And so I met with a group of patient partners in Toronto, and we prioritized the projects [based on] what we thought would be most beneficial to us as patients. And we were fortunate in that there were three Indigenous projects that actually got prioritized in the top. It’s very important research for us as Indigenous people, especially because of the high incidence of kidney disease and diabetes within our communities.

Now I sit on the leadership team, and I will say that has been something that I didn’t feel was in my lane, but I have thoroughly enjoyed it, and I welcome the opportunity. I appreciate having a voice at the leadership table, and . . . [using] my voice to let the doctors, researchers, the executive director, and policy-makers know how I feel about the direction or how things are happening, or what we can change, to be able to better the experiences and outcomes for patients.

We’ve given patients a voice to be involved in their care and their family’s care throughout their kidney journey and I think that’s really important. To be able to see these changes in my lifetime about kidney disease is so amazing . . . to be able to see the changes and see good things happen, it’s just so exciting to be part of that.

Researchers are data driven, and we at Can-SOLVE have proved that [we can generate data]. But we have also improved research through storytelling and the patient voice providing the qualitative insight, which is very important, and I believe, far more important than what only the numbers say. For [patient partners] to be involved in their own community and their own region, to be able to be part of this knowledge mobilization effort is vitally important.
